# Models for age-specific estimation of appendicular skeletal muscle mass using the ultrasound-measured rectus femoris muscle thickness

**DOI:** 10.18632/aging.206294

**Published:** 2025-08-01

**Authors:** Ga Yang Shim, Jong Bum Kim, Chang Won Won, Jae-Young Lim

**Affiliations:** 1 Department of Physical and Rehabilitation Medicine, Kyung Hee University Hospital, Seoul, Republic of Korea; 2 Department of Rehabilitation Medicine, TK Orthopedic Surgery Hospital, Gyeonggi, Republic of Korea; 3 Department of Family Medicine, Kyung Hee University College of Medicine, Kyung Hee University Hospital, Seoul, Republic of Korea; 4 Department of Rehabilitation Medicine, Seoul National University Bundang Hospital, Seoul National University College of Medicine, Gyeonggi, Republic of Korea; 5 Institute on Aging, Seoul National University, Seoul, Republic of Korea

**Keywords:** ultrasound, muscle thickness, appendicular muscle mass, bioelectrical impedance analysis, equation

## Abstract

Ultrasound is a useful tool for assessing muscle status. Estimation equations based on ultrasound measurements have been used to approximate appendicular skeletal muscle mass (ASM). However, age-related changes in skeletal muscle may influence the accuracy of ultrasound-based measurements, as compared to other established techniques. This study aimed to examine these associations across various age groups and to determine whether age-specific models are required for ASM estimation. A total of 265 subjects were analyzed and divided into three age groups: young (Group A, *n* = 94), middle-aged (Group B, *n* = 84), and older (Group C, *n* = 87). Rectus femoris (RF) muscle thickness (MT) was measured using ultrasound and ASM assessed using bioelectrical impedance analysis, which served as the reference method. Multivariate linear regression models were developed for each age group and for total group (Groups A+B+C) using RF MT as the primary predictor. All models showed high adjusted R^2^ values (0.881–0.955). Group-specific models demonstrating greater accuracy than total group model, based on lower root mean square error, the mean absolute error, and higher adjusted R^2^. These findings highlight the clinical relevance of using group-specific models to enhance the accuracy of ultrasound-based ASM estimation, thereby improving the screening and early identification of sarcopenia. Future validation in diverse populations and clinical settings is warranted.

## INTRODUCTION

Sarcopenia, a geriatric syndrome characterized by a loss of muscle mass and function, is associated with adverse clinical outcomes such as falls, mobility limitation, hospitalization, and mortality [1–3]. The prevalence varies by the definition thereof, but sarcopenia incidence increases with age [4]. As Korea is among the fastest-aging countries worldwide, there is an urgent need for simple and practical tool to evaluate sarcopenia in clinical settings.

Quantification of muscle mass is a key sarcopenia diagnostic criterion [5]. Although advanced imaging modalities such as computed tomography, magnetic resonance imaging, and dual-energy x-ray absorptiometry are considered the gold standards when assessing muscle mass, their high cost, limited accessibility, and challenging technical requirements render them impractical for routine clinical use. Bioelectrical impedance analysis (BIA) has emerged as a cost-effective alternative. However, its accuracy can be compromised by factors such as hydration status and bodily composition [6, 7].

Ultrasound has attracted increasing attention as a portable, non-invasive, and accessible tool for assessment of muscle quantity and quality. Of the various ultrasound measurements, rectus femoris (RF) muscle thickness (MT) has been the most extensively studied [8] and has been suggested to serve as a proxy of appendicular skeletal muscle mass (ASM) [9]. Nevertheless, the validity of RF MT as a predictor of ASM may be influenced by age-related changes in skeletal muscle. These include fiber-type transitions (selective loss of type 2 fibers) and intramuscular fat infiltration (myosteatosis) [10–12]. Such physiological alterations can modify ultrasound signal propagation and tissue echogenicity, potentially weakening the association between RF MT and muscle mass.

While several ultrasound-based equations for ASM estimation have been proposed in previous studies, few have been externally validated across different age groups or ethnic populations [13]. Therefore, this study aimed to develop equation models for ASM estimation using RF MT in three distinct age groups (young, middle-aged, and older adults), as well as in the total group. We further evaluated whether group-specific models improved the accuracy of ASM estimation compared to total group model.

## RESULTS

A total of 265 subjects were included in this study. They were divided into three age groups: young adults (Group A, 20–39 years, *n* = 94), middle-aged adults (Group B, 40–59 years, *n* = 84), and older adults (Group C, 70–89 years, *n* = 87). The group characteristics are summarized in Table 1. There were significant among-group differences in all measured parameters except sex and the BMI. On post-hoc analysis, Group C was older, all of their height, weight, ASM, and RF MT were lower than those of Groups A and B. There was no significant difference in muscle mass (ASM or RF MT) between Groups A and B, but Group A exhibited a higher ASM and a greater RF MT.

**Table 1:** Characteristics of subjects according to age groups.

	**Group A (*N* = 94)**	**Group B (*N* = 84)**	**Group C (*N* = 87)**	***P*-value**	**Post hoc**
Age, years	29.5 ± 4.9	48.6 ± 5.7	81.0 ± 5.1	<0.001	1,2,3
Sex (M:F)	49:45	41:43	37:50	0.199	
Height, cm	169.7 ± 8.9	165.7 ± 8.9	158.6 ± 8.6	<0.001	1,2,3
Weight, kg	64.8 ± 11.5	62.4 ± 12.4	57.2 ± 9.7	<0.001	2,3
BMI, kg/m^2^	22.4 ± 2.8	22.5 ± 2.9	22.6 ± 2.9	0.808	
ASM, kg	5.3 ± 0,5	5.1 ± 0.6	3.8 ± 0.4	<0.001	2,3
RF MT, cm	1.9 ± 0.4	1.8 ± 0.4	1.2 ± 0.4	<0.001	2,3

Post-hoc analysis: 1, Group A versus Group B; 2, Group A versus Group C; 3, Group B versus Group C. Abbreviations: MBI: body mass index; ASM: appendicular skeletal muscle mass; RF: rectus femoris; MT: muscle thickness.

Table 2 shows the effects of age, sex, height, weight, and RF MT on the ASM. The ASM did not correlate with age in any age group but significantly decreased with age in the total group. The ASM exhibited strong positive correlations with sex (1 for men, 0 for women), height, and weight, and a weak-to-moderate correlation with the ultrasound-measured RF MT, depending on the age group.

**Table 2:** The effect of age, sex, height, and weight, rectus femoris muscle thickness on appendicular skeletal muscle mass.

	**Group A (20–39 years)**	**Group B (40–59 years)**	**Group C (70–89 years)**	**Total group (20–89 years)**
Age	0.059	−0.264^*^	−0.108	−0.454^**^
Sex†	0.783^**^	0.678^**^	0.608^**^	0.741^**^
Height	0.872^**^	0.911^**^	0.882^**^	0.865^**^
Weight	0.883^**^	0.912^**^	0.783^**^	0.903^**^
RF MT	0.421^**^	0.610^**^	0.376^**^	0.608^**^

^†^Spearman correlation. ^*^P < 0.05, ^**^P < 0.01. Abbreviations: RF: rectus femoris; MT: muscle thickness.

Based on correlation analysis, multivariate linear regression was performed to estimate ASM using RF MT as the main independent variable (Supplementary Table 1). The models were:

*ASM estimation model for Group A* (*kg*) = 1.398 × *RF MT* (*cm*) + 0.222 × *height* (*cm*) + 0.192 × *weight* (*kg*) + 2.625 × *Sex* (*Men* = 1, *Women* = 0) − 32.799
*ASM estimation model for Group B* (*kg*) = 1.501 × *RF MT* (*cm*) + 0.232 × *height* (*cm*) + 0.154 × *weight* (*kg*) + 2.166 × *Sex* (*Men* = 1, *Women* = 0) − 31.754
*ASM estimation model for Group C* (*kg*) = 1.012 × *RF MT* (*cm*) + 0.217 × *height* (*cm*) + 0.143 × *weight* (*kg*) + 1.447 × *Sex* (*Men* = 1, *Women* = 0) − 28.794
*ASM estimation model for total group* (*kg*) = 1.012 × *RF MT* (*cm*) + 0.217 × *height* (*cm*) + 0.143 × *weight* (*kg*) + 1.447 × *Sex* (*Men* = 1, *Women* = 0) − 0.019 × *Age* − 28.503

**Figure 1:**
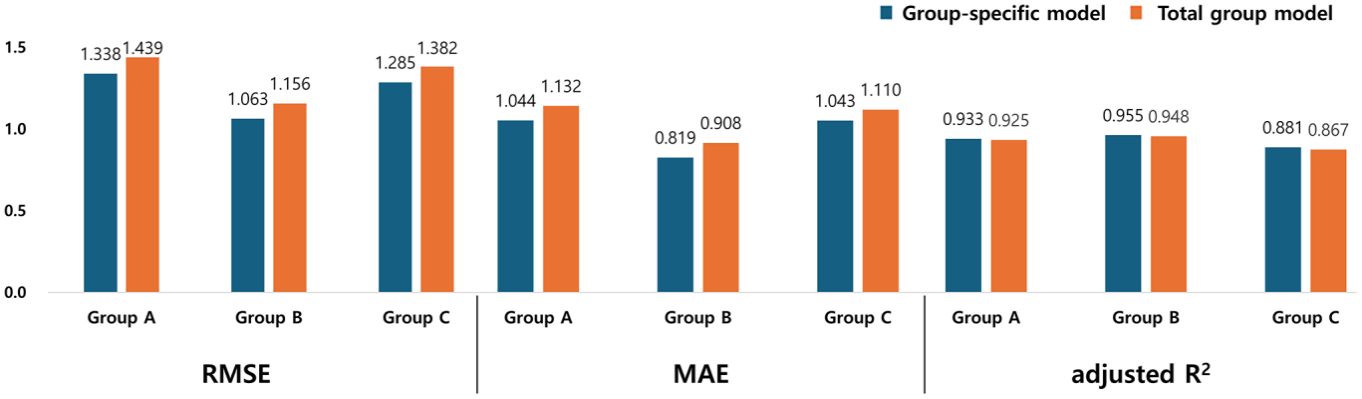
**Comparison of root mean square errors, mean absolute errors, and adjusted R^2^ between group-specific and total group regression models for ASM estimation.** Bar graphs display model performance metrics—root mean square error (RMSE), mean absolute error (MAE), and adjusted R^2^—across three age groups (Group A: young adults, Group B: middle-aged adults, Group C: older adults). For each group, values from the group-specific regression model (blue) and the total group model (orange) are compared. Across all three metrics, group-specific models demonstrated consistently better performance than the total group model, with lower RMSE and MAE values and higher adjusted R^2^ across all groups. These findings suggest that group-specific models improve the accuracy of ASM estimation by accounting for age-related muscle changes. Abbreviations: RMSE: root mean square error; MAE: mean absolute error; R^2^: coefficient of determination; ASM: appendicular skeletal muscle mass.

To compare the accuracy of models, the root mean square error (RMSE), mean absolute error (MAE), and adjusted R^2^ values are presented in Figure 1 and Table 3. The group-specific models consistently exhibited lower RMSE and MAE values across all groups, indicating smaller estimation errors compared to the total group model. Similarly, adjusted R^2^ values were consistently higher in the group-specific models, demonstrating greater explanatory power in ASM.

**Table 3:** Comparison between performance metrics of all models.

	**RMSE**			**MAE**			**Adjusted R^2^**		
	**Group A**	**Group B**	**Group C**	**Group A**	**Group B**	**Group C**	**Group A**	**Group B**	**Group C**
Group-specific model	1.338	1.063	1.285	1.044	0.819	1.043	0.933	0.955	0.881
Total group model	1.439	1.156	1.382	1.132	0.908	1.110	0.925	0.948	0.867

Abbreviations: RMSE: root mean square error; MAE: mean absolute error.

**Figure 2:**
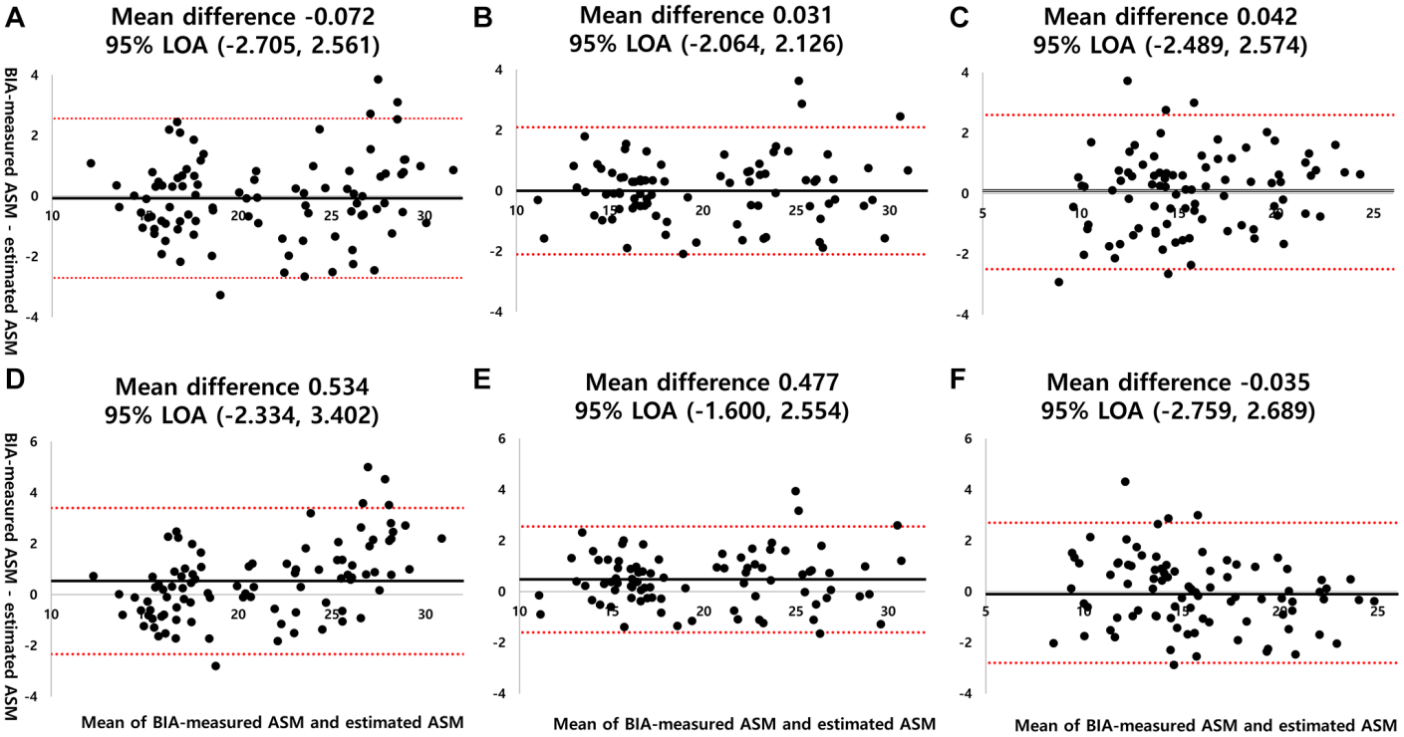
**Bland-Altman plots for estimated ASM and BIA-measured ASM across groups and models.** The solid and dashed lines represent the mean difference and 95% LOA, respectively. (**A**) Group-specific model derived from Group A applied to Group A, (**B**) group-specific model derived from Group B applied to Group B, (**C**) group-specific model derived Group C applied to Group C, (**D**) total group model applied to Group A, (**E**) total group model applied to Group B, (**F**) total group model applied to Group C. Abbreviations: BIA: bioelectrical impedance analysis; ASM: appendicular skeletal muscle mass; 95% LOA: 95% limit of agreement.

The Bland–Altman plots show the agreement between the measured and estimated ASM values (Figure 2). The plots reveal that the mean differences were small, most data were within the 95% agreement, and no evidence of systematic errors.

## DISCUSSION

This study developed equations that estimated the ASM using RF, and compared the accuracy of the group-specific models and total group model for ASM estimates. Based on errors (RMSE and MAE) and explanatory power (adjusted R^2^), the group-specific model provided greater accuracy ASM estimates than the total group model in all three groups. These findings suggest the importance of employing group-specific models stratified by age that consider diverse muscle characteristics when estimating the ASM.

Ultrasound is a valuable tool for muscle assessment, and its potential role in sarcopenia diagnosis has been increasingly recognized [14]. Previous studies reported moderate correlations between RF MT in ultrasound data and ASM [15, 16], with a pooled correlation coefficient of 0.56 according to a recent meta-analysis [17]. In line with these findings, our study confirmed a significant correlation between RF MT and ASM. However, the strength of this correlation differed by age group - strongest in middle-aged adults (Group B, r = 0.610), followed by young adults (Group A, r = 0.421), and weakest in older adults (Group C, r = 0.376). This age-related variability may reflect underlying structural and metabolic changes in skeletal muscle with aging. In older adults, increased intramuscular fat accumulation reduces the actual muscle mass, which is not accurately reflected in RF MT measured using ultrasound [18]. Furthermore, aging selectively reduces the proportion of type 2 fibers; that of type 1 fibers increases. As the RF is composed predominantly of type 2 fibers, RF atrophy disproportionately affects the contribution thereof to the ASM, and therefore weakens the relationship between the RF MT and ASM in older adults [11, 19].

The European Working Group on Sarcopenia recommended measuring ASM to identify a low muscle mass [20]. Accordingly, previous studies developed equations to estimate ASM using ultrasound measurements. Abe et al. derived models predicting ASM using MT data from seven sites including the upper and lower limbs and trunk (for Caucasian) and four sites including the upper and lower limbs (for Japanese) [21, 22]. Paris et al. developed a four-site ultrasound protocol that quantified ASM at the bedside by measuring the bilateral RF MT and vastus intermedius MT [23]. Barbosa-Silva et al. presented equations that combined the ultrasound-measured MTs of the dominant arm and thigh with anthropometric variables when estimating ASM [24]. Baek et al. proposed ASM estimation equations for Koreans using a combination of ultrasound-measured MT and echo intensity of the biceps and RF from the dominant side [25]. However, ultrasound measurements of multiple bodily sites are time-consuming and therefore not feasible in routine clinical practice. To address this, Abe et al. proposed an equation to estimate ASM using single-site forearm MT [21, 26].

Our study measured a single muscle measurement, RF, for ASM estimation equations, which achieved higher adjusted R^2^ and lower standard error of the estimate (SEE) than studies using multiple measurement sites or other muscle groups [24, 25]. The RF muscle is an ideal surrogate when detecting age-related changes in muscle mass because the RF is prone to selective loss on aging [11, 27]. Moreover, due to its superficial anatomical location, the RF is more reliably visualized and measured via ultrasound than deeper muscle (attenuation). This renders the RF particularly appropriate as a measurement site in subjects with thick musculatures, such as young men, since assessing a single muscle such as the RF is easier than a muscle group such as the quadriceps femoris.

Several previous studies have developed ASM estimation equations for specific age groups, particularly in older adults [22, 24, 28]. A recent systematic review raised critical questions regarding whether age should simply be included as a covariate in the model, or whether entirely separate equations should be developed for different age groups [13]. Our findings support the latter: when comparing RMSE, MAE, and adjusted R^2^, group-specific models consistently showed greater accuracy than the total group model across all age groups. This suggests that the age-related changes in muscle structure and composition, such as fat infiltration, fiber-type shifts, and hydration variability, are non-linear and cannot fully account for by including age as a linear variable in total group model [10, 11, 29].

Group C consisted of subjects who performed muscle evaluation as part of the diagnosis and management of sarcopenia. It is possible that subjects with preexisting muscle deterioration were included, as the mean score of Strength, Assistance with walking, Rise from a chair, Climb stairs and Falls (SARC-F) in Group C was 4.7, exceeding the diagnostic criterion of 4. Consequently, this group may not fully represent the general population of community-dwelling older adults. Similarly, subjects in their 60s were excluded due to the heterogeneity of muscle assessment data and the small sample size. Subjects in their 60s who undergo sarcopenia evaluation in hospital are relatively rare and their mean SARC-F score was 6.7, higher than the Group C, suggesting more advanced functional impairment and possibly undiagnosed conditions. Therefore, this exclusion was necessary to improve homogeneity.

This study had several limitations. First, ASM was measured by BIA served as the reference variable. BIA analyzes body composition using multi-frequency impedance and then estimates ASM by applying a specific equation derived from dual-energy X-ray absorptiometry, which is considered the gold standard. Thus, accuracy of BIA may be affected by hydration status and bodily composition, particularly in older adults [30, 31]. On the other hand, DXA offers higher accuracy but is limited by higher cost, accessibility, and radiation exposure, which reduces its feasibility in routine clinical settings. Therefore, while the regression models developed in this study using BIA is clinically applicable, further validation against DXA-derived ASM values is required. Second, the study exhibited methodological heterogeneity in data collection, subjects, and devices between the two datasets. The young and middle-aged groups were recruited in a research setting, whereas the data on older adults were derived from a retrospective cohort study performed in a clinical setting. In addition, although all devices satisfy the quality control standards and they were used according to standardized protocols, no cross-device calibrated method was employed, which may introduce potential bias. Third, the models were developed exclusively in the Korean population. Given potential ethnic differences in muscle distribution, body composition, and age-related muscle changes the generalizability of these models may be limited. Therefore, external validation in diverse populations, including Western and other ana-Asian cohorts, is necessary. Finally, the ecological validity of models using ultrasound-based RF MT in non-specialist clinical settings may be limited by operator experience and complexity of the model. Nonetheless, the RF is a superficial muscle with well-defined anatomical landmarks, previous studies have shown that even non-experts can obtain reliable measurements when trained using a standardized protocol [32–34].

Despite these limitations, the study demonstrated that ASM can be reliably estimated using ultrasound-derived RF MT in Korean adults across various age groups. Notably, group-specific models provide important advantages for more accurately estimating ASM across age groups. These findings support the use of group-specific models using single muscle ultrasound measurements for clinical assessment and early detection of sarcopenia, particularly in primary care and rehabilitation settings. To enhance generalizability, future research should externally validate these models in diverse ethnic background and clinical settings.

## METHODS

### Study population and design

This study used two datasets with different recruitment settings and objectives. First, dataset of young and middle-aged adults was derived from a prospective, stratified recruitment by age, sex, and phenotype (e.g., fast-twitch, slow-twitch exercise, control and low body mass index group) of 180 general population aged 20–59 years at Kyung Hee University Hospital between December 2021 and December 2022 as part of a government-funded research project for medical device development and validation. Muscle assessments, including ultrasound and BIA, were performed in a controlled research conditions with consistent protocol. Second, the older adult dataset (aged ≥60 years; *n* = 126) was derived from a retrospective clinical cohort at the Department of Rehabilitation Medicine, Seoul National University at Bundang Hospital between March 2020 and November 2023. The subjects were patients referred for sarcopenia evaluation and management as part of routine clinical practice, thus were more likely to have preexisting muscle loss or comorbidities. Ultrasound and BIA assessments were performed based on clinical indications, and data were extracted from electronic medical records.

**Figure 3:**
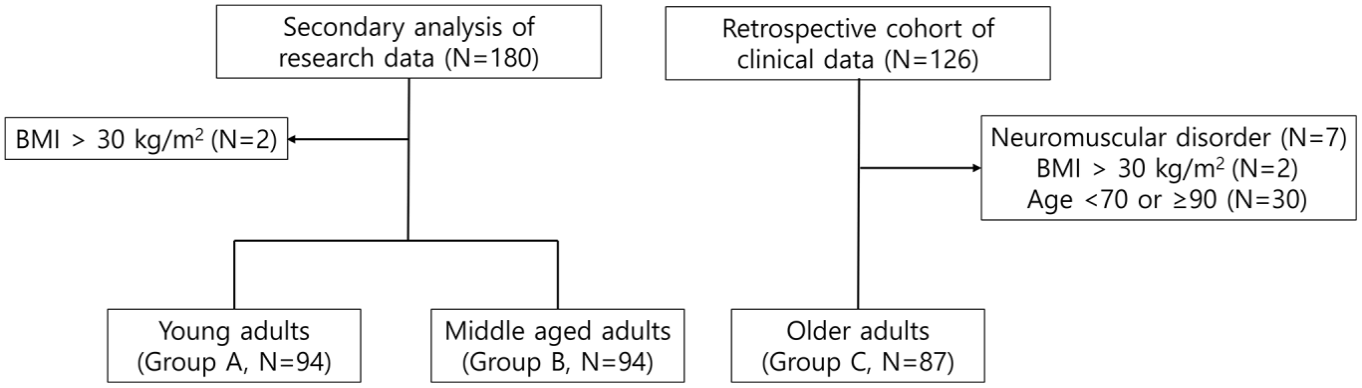
Flowchart of the subjects.

Subjects were divided into three age groups: Young adults (Group A, 20–39 years), middle-aged adults (Group B, 40–59 years), and older adults (Group C, 70–89 years). This classification was based on distinct physiological differences in both muscle mass and function across the lifespan, with young adults (20–30 years) exhibiting peak muscle mass; middle-aged adults (40–50 years) evidencing the onset of muscle loss; and older adults (70–80 years) exhibiting the most pronounced losses of muscle mass, strength, and function [18, 19]. Subjects in their 60s due to significant data heterogeneity and limited sample size, and those in their 90s were excluded due to advanced age and the potential for pathological conditions. Additionally, those with neuromuscular disorders (e.g., radiculopathy, poliomyelitis, myopathy), mobility limitation (e.g., stroke, cerebral palsy, recent orthopedic surgery), amputations, or body mass index (BMI) ≥30 kg/m^2^ were excluded as these factors can affect muscle integrity or compromise the accuracy of BIA results [7, 35]. The detailed study flow is outlined in Figure 3.

The study was conducted in accordance with the ethical standards of the Declaration of Helsinki, and all procedures were approved by the Institutional Review Board (IRB) of the Clinical Research Ethics Committees of the Kyung Hee University Medical Center (IRB number: KHUH 2021-04-065-061) and Seoul National University at Bundang Hospital (IRB number: B-2407-912-111). Written informed consent was obtained from all subjects.

### Skeletal muscle mass measurement

ASM was measured using BIA (InBody 770 for Group A and B and InBody S10 for Group C; InBody Inc., Seoul, Korea). Subjects were asked to wear minimal clothing and no jewelry, metal, or shoes. With the InBody 770 measured in a standing position and the InBody S10 in a supine position, a total of 30 impedance values were obtained across 5 body segments (right and left arms, right and left legs, and trunk) at 6 different frequencies (1, 5, 50, 250, 500, and 1,000 kHz). The ASM was the combined skeletal muscle masses of the arms and legs.

For Group A and B, BIA was performed by a research nurse, for Group C, by a clinical nurse. In all cases, BIA was performed prior to the ultrasound assessment and the ultrasound operators were not intentionally informed of the BIA results.

### Ultrasound measurements

For Group A and B, two trained graduate students acquired ultrasound images using the LOGIQ 200 PRO system (GE Medical Systems, Milwaukee, WI, USA) with a 7.5-MHz LH linear transducer. All images from these groups were subsequently analyzed by one of the study authors (G.Y.S) using ImageJ software (NIH, Bethesda, MD, version 1.6.0_24). For Group C, image acquisition and analysis using on-screen calipers were performed by four physiatrists with more than 3-year of experience in musculoskeletal ultrasound, using the Affiniti 70G system (Philips, Amsterdam, the Netherlands) with an 18-MHz linear transducer.

RF was assessed using a standardized protocol [36]. Longitudinal ultrasound images were obtained by placing the probe parallel to the muscle fiber at the midpoint between the proximal border of the femur and patella, with the subject in a relaxed supine position and the knee fully extended. MT was defined as the vertical distance between the superficial and deep aponeurosis, measured at the center of the image. All images were acquired three times by repositioning the probe for each acquisition, and a single measurement was performed on each image. Acquisitions were conducted without compression following generous application of ultrasound gel. Intra-rater reliability showed excellent consistency, with intra-class correlation coefficients exceeding 0.90 across all groups.

### Statistical analysis

Data on the three groups were compared using analysis of variance for continuous variables and chi-squared test for categorical variables. Correlations between variables were assessed using Pearson correlation coefficients for continuous variables and Spearman correlation coefficients for categorical variables.

Multivariate linear regression was performed to derive equation models for each group and the total group (Group A+B+C). The main independent variable was RF MT and the dependent variable was ASM. Covariates were selected based on correlations in previous studies and this study. Stepwise selection method applied in each model.

The SEE and the coefficient of linear determination (R^2^) were calculated. Multicollinearity was assessed using the variance inflation factor (VIF), with a threshold of VIF >10 indicating significant collinearity; all VIF values were within acceptable limits. The extent of agreement between measured and estimated ASMs was assessed by drawing Bland-Altman plots. The accuracy of the group-specific models and total model were evaluated by comparing the RMSE, MAE, and the adjusted R^2^ across the three groups. Last, to assess the validity of the models, we performed 10000 replicate bootstrapping. The bootstrap-estimated RMSE for each group-specific model was 1.333 kg in Group A, 1.057 kg in Group B, and 1.279 kg in Group C, respectively, differing by less than 0.005–0.006 kg from the original RMSE, indicating low risk of overfitting.

All statistical analyses were performed using IBM SPSS software (version 23.0 for Windows; IBM Corp., Armonk, NY, USA). *P*-values <0.05 were considered statistically significant.

## Supplementary Materials

Supplementary Table 1
