# Supplementary material for: Models for age-specific estimation of appendicular skeletal muscle mass using the ultrasound-measured rectus femoris muscle thickness
Source: Aging (Albany NY). 2025 Aug 1;17(8):1988–98. doi: 10.18632/aging.206294 (PMC12422818; doi:10.18632/aging.206294)
Supplement: Supplementary Table 1 [file aging-17-8-206294-s001.pdf]

SUPPLEMENTARY TABLE

Supplementary Table 1. Multivariate linear regression model for estimating appendicular skeletal muscle mass.

	Unstandardized β ± standard error			
	**Group A (20–39 years)**	**Group B (40–59 years)**	**Group C (70–89 years)**	**Total group (20–89 years)**
Intercept	−32.799 ± 4.184	−31.754 ± 3.563	−28.794 ± 4.163	−28.503 ± 2.645
RF MT (cm)	1.398 ± 0.404	1.501 ± 0.362	1.012 ± 0.413	1.382 ± 0.235
Height (cm)	0.222 ± 0.028	0.232 ± 0.025	0.217 ± 0.030	0.209 ± 0.017
Weight (kg)	0.192 ± 0.020	0.154 ± 0.018	0.143 ± 0.020	0.174 ± 0.012
Sex (men = 1, women = 0)	2.625 ± 0.434	2.166 ± 0.381	1.447 ± 0.448	2.170 ± 0.256
Age (years)	–	–	–	−0.019 ± 0.005
adjusted R^2	0.933	0.955	0.881	0.937
SEE	1.373	1.096	1.315	1.349

Abbreviations: RF: rectus femoris; MT: muscle thickness; SEE: standard error of the estimate.
